# Beyond Cholera: Characterization of *zot*-Encoding Filamentous Phages in the Marine Fish Pathogen *Vibrio anguillarum*

**DOI:** 10.3390/v12070730

**Published:** 2020-07-06

**Authors:** Jesper Juel Mauritzen, Daniel Castillo, Demeng Tan, Sine Lo Svenningsen, Mathias Middelboe

**Affiliations:** 1 Marine Biological Section, University of Copenhagen, Strandpromenaden 5, 3000 Helsingør, Denmark; jesper.mauritzen@bio.ku.dk (J.J.M.); daniel.castillo@bio.ku.dk (D.C.); 2 Section for Biomolecular Sciences, University of Copenhagen, Ole Maaløes Vej 5, 2200 København N, Denmark; demengtan@gmail.com (D.T.); sls@bio.ku.dk (S.L.S.)

**Keywords:** filamentous phage, *Vibrio anguillarum*, *zot*, horizontal gene transfer, marine environment

## Abstract

Zonula occludens toxin (Zot) is a conserved protein in filamentous vibriophages and has been reported as a putative toxin in *Vibrio cholerae*. Recently, widespread distribution of *zot-*encoding prophages was found among marine *Vibrio* species, including environmental isolates. However, little is known about the dynamics of these prophages beyond *V. cholerae*. In this study, we characterized and quantified the *zot-*encoding filamentous phage VAIϕ, spontaneously induced from the fish pathogen *V. anguillarum*. VAIϕ contained 6117 bp encoding 11 ORFs, including ORF8^pVAI^, exhibiting 27%–73% amino acid identity to *Inovirus* Zot-like proteins. A qPCR method revealed an average of four VAIϕ genomes per host genome during host exponential growth phase, and PCR demonstrated dissemination of induced VAIϕ to other *V. anguillarum* strains through re-integration in non-lysogens. VAIϕ integrated into both chromosomes of *V. anguillarum* by recombination, causing changes in a putative ORF in the phage genome. Phylogenetic analysis of the *V. anguillarum* *Inoviridae* elements revealed mosaic genome structures related to mainly *V. cholerae*. Altogether, this study contributes to the understanding of *Inovirus* infection dynamics and mobilization of *zot*-like genes beyond human pathogenic vibrios, and discusses their potential role in the evolution of the fish pathogen *V. anguillarum*.

## 1. Introduction

The *Inovirus* genus comprises filamentous phages encapsulating circular (+) ssDNA genomes. In contrast to dsDNA tailed phages, filamentous phages do not lyse their host cell upon propagation, but are secreted through the host membrane in an infection process called chronic cycle [1]. In gram-negative hosts, filamentous phages attach to pili that are thought to spontaneously retract resulting in the entry of the phage genome into the host cytoplasm [1,2]. Following host entry, filamentous phage genomes replicate using the bacterial host machinery to synthesize the (−) strand, producing a circular dsDNA phage genome, usually referred to as the replicative form, which serves as a template for rolling circle replication and transcription of phage genes [1]. Filamentous phage genomes are usually 5–20 kb long, organized in replication, structure and assembly modules [1,3,4]. Due to foundational research in the *Escherichia coli* Ff phages and the *V. cholerae* phage CTXϕ, genome structures of filamentous phages can now be compared and their functions predicted [1,4].

The vast majority of characterized filamentous phages can integrate their genome into the host chromosome. Some filamentous phages encode an integrase, resolvase or transposase that likely allow genome insertions into the bacterial chromosome [4,5,6,7]. However, filamentous vibriophages containing *dif*-like (*attP*) sites integrate by utilizing the host-encoded XerCD recombinases that normally resolve chromosome dimers at *dif* sites [1,8]. Subsequent prophage excision contributes the establishment of the replicative form and has been shown to occur either spontaneously or in a RecA-dependent manner for the filamentous vibriophages VGJϕ and CTXϕ, respectively [8,9,10]. Secretion of the filamentous phages through the bacterial membrane may be facilitated by a phage- or host-encoded secretin [1,7,11] and the production rates vary greatly within the *Inovirus* genus, with CTXϕ reaching titers of 10^5^–10^7^ particles mL^−1^, and *E. coli* phages rising up to 10^12^ virions mL^−1^ during in vitro cultivation [12,13]. The interaction between filamentous phages and their hosts is often considered a symbiotic relationship, where phage replication only modestly burdens the host, and in return contributes to host virulence and evolution [4]. For example, lysogenic conversion of *V. cholerae* has been reported for the filamentous phage CTXϕ that mobilizes the genes encoding the cholera toxin, which is one of the main responsible factors for the outbreak of cholera [13]. Besides the cholera toxin, CTXϕ encodes the Zonula occludens toxin (Zot) among other potential virulence factors [13]. Specifically, the Zot protein seems to possess a dual function as it is essential for CTXϕ morphogenesis [13] but has also been reported to display enterotoxic activity, and it is thus hypothesized to contribute to the classic diarrhea symptom of *V. cholerae* infections [14,15]. Contrarily, integration of an *Inovirus* genome into the bacterial chromosome in *Xanthomonas axonopodis* inhibited the growth, reduced the motility and caused the loss of virulence of this pathogen [16]. Apart from mobilizing genes, filamentous phage particles have been reported to induce phenotypic diversity in *Pseudomonas aeruginosa* biofilms and increase the biofilm stability and survival of the cells [6,17]. Thus, filamentous phages influence their host by various means and while several findings suggest that *Inoviridae* phages play important roles involved in human and animal diseases [14,18,19,20,21,22], there are still many open questions related to the role of these viruses for bacterial virulence. Indeed, prophages belonging to the *Inoviridae* family are extensively present in both clinical and environmental isolates; thus, these phages are not exclusively associated with pathogens [3,23]. However, the widespread distribution of *Inoviridae*-encoded virulence-associated genes in both deep sub-seafloor sediments [24] and in other environmental *Vibrio* bacteria [23] suggests that the oceans may serve as a genetic reservoir for such genes. Therefore, an improved understanding of the biology and consequences of the specific phage-bacteria interactions and their potential role in dissemination of diseases in the marine environment is required. 

Besides CTXϕ, only fourteen genomes of described inoviruses, all infecting human pathogenic *Vibrio* species, have been deposited in the NCBI database. Thus, despite their global distribution and presence as prophages in >45% of environmental *Vibrio* species [23], our understanding of filamentous vibriophages beyond human pathogens is very limited. One of the important environmental vibrios that carries *Inovirus* prophages is *V. anguillarum*, a marine fish pathogen that causes vibriosis, a hemorrhagic septicemic disease responsible for the demise of more than 50 fish species of industrial importance [25]. Recent studies predicted the *zot*-like genes to reside on prophage elements in three *V. anguillarum* strains isolated from Chile, the UK and the US, respectively [26], and suggested that the loss of a *zot*-encoding prophage reduced virulence against fish larvae in a pathogenic *V. anguillarum* [27]. These results suggest that *Inovirus*-related prophages may spread in *V. anguillarum* and that inoviruses could contribute to the virulence of this bacterium, as proposed for the related species *V. cholerae* and *V. parahaemolyticus* [13,14,23,28]. 

The aim of this study was to explore the dynamics of this specific group of *zot*-encoding prophages in *V. anguillarum* and their potential as vehicles for dispersal of potential virulence factors in the marine environment. This work reveals the presence and genome sequences of novel inoviruses infecting the fish pathogen *V. anguillarum* and discusses their possible contribution to lysogenic conversion and evolution of this fish pathogen. 

## 2. Materials and Methods 

### 2.1. Vibrio anguillarum Strains, Medium Composition and Growth Conditions

The *V. anguillarum* strains used in this study were sequenced previously (Table 1) [26] and were preserved at −80 °C in MB (marine broth: 5 g tryptone, 1 g yeast extract and 20 g sea salts in 1 L of Milli-Q H_2_O) with 35% glycerol. The strains were inoculated from −80°C stocks onto MB agar plates (MB with 1.5% Agar) and cultured at room temperature for a minimum of 24 h prior to further analysis [29]. Unless otherwise specified, *V. anguillarum* strains were cultured under standard conditions: liquid MB, 100 rpm aeration and at room temperature. 

### 2.2. Stability of zot-Encoding Prophages

To investigate the stability of the *zot*-encoding prophages, the *V. anguillarum* strains PF4, T265 and Ba35 were inoculated from −80°C freezer stocks in MB for 24 h. Then, each culture was serially diluted, spread onto MB agar plates and incubated at room temperature for up to 2 days. After incubation, 50 colonies of each strain were individually inoculated in MB for at least 4 h. Subsequently, the culture was used as a template for PCR, targeting the prophage-related gene, *zot*, to evaluate the presence of *zot*-encoding prophages in the individual isolates (Table S1). Conventional PCR analysis was carried out using MyTaq™ Red DNA Polymerase (Bioline, Taunton, MA, USA).

### 2.3. Detection of Circularized Prophages

To evaluate whether the *zot*-encoding prophages retained in the *V. anguillarum* strains PF4*-zot-*prophage^+^ (PF4-derived strain containing the *zot*-encoding prophage, Table 1), T265 and Ba35 existed in a circularized form, and primers oppositely directed at the prophage boundaries were designed such that a detectable PCR product of an expected size occurred only if the element was circularized (Figure S1; Table S1). Briefly, one colony from the *V. anguillarum* strains PF4*-zot-*prophage^+^, T265 and Ba35 were cultured under standard conditions overnight and 1 µL of the culture was used as a template for PCR as described above.

### 2.4. Evaluation of Prophage Induction by Detection of Nuclease-Resistant zot in the Supernatant

The presence of inoviruses in the supernatants of the *V. anguillarum* strains PF4*-zot-*prophage^+^, T265 and Ba35 was evaluated as previously described [7]. Briefly, cell-free supernatants with and without DNase I treatment were used as a template for PCR, using primers targeting a phage gene*, zot* and a bacterial chromosomal reference gene: *dnaJ* (Table S1). PCR analysis that showed the presence of *zot* and absence of *dnaJ* in the DNase I treated samples was interpreted as the presence of *Inovirus* particles.

### 2.5. Buoyant Density of VAIϕ 

The buoyant density of the *zot*-encoding elements (VAIϕ) was examined by cesium chloride (CsCl) gradients of mitomycin C treated cultures. *V. anguillarum* strains PF4 and T265 were cultured in MB overnight. Then, the cultures were diluted 1:3 in MB +/− mitomycin C (final concentration: 50 ng mL^−1^: Sigma-Aldrich, St. Louis, MO, USA) and incubated under standard conditions for 24 h. Supernatants were 0.2 µm-filtered and checked for sterility by plating. The supernatants of each strain were concentrated by ultracentrifugation in Polyallomer centrifuge tubes (Beckman Coulter, Brea, CA, USA) in a Beckman Optima™ LE-BOK Ultracentrifuge using the SW 55 TI rotor at 40,000 rpm for 1 h at 4 °C. Then, the upper 80% of the supernatant was discarded, the tubes were refilled with supernatant and centrifuged as described above. This step was repeated until a total of 102 mL supernatant was concentrated to 6 mL. Then, 1 mL of the supernatant concentrates was loaded onto gradients containing fractions of 1.2–1.6 g mL^−1^ CsCl and centrifuged at 50,000 rpm for 20 h at 4 °C. Finally, fractions were collected in 0.4 mL steps using a 27 G needle (BRAUN, Melsungen, Germany) and the densities were determined geometrically. Each fraction was then diluted tenfold and 1 µL was used as a template in a PCR reaction targeting *zot* (Table S1). 

### 2.6. Transmission Electron Microscopy

To examine the morphology of VAIϕ particles, a culture of *V. anguillarum* strain Ba35 was prepared for electron microscopy using the prophage-negative strain *V. anguillarum* PF4-*zot*-prophage^−^ (Table 1) as a negative control. Both strains were cultured under standard conditions overnight, mounted on Formvar-coated 200 mesh copper grids, and then negatively stained with 2% phosphotungstic acid and examined in a JEM-1010 transmission electron microscope (Jeol, Tokyo, Japan). Filaments with a width of 7 nm and 15–20 nm were interpreted as filamentous phages and flagella, respectively, as measured by ImageJ (v.1.52a). 

### 2.7. Genomic Sequencing of pVAIs

The replicative forms in *V. anguillarum* strains PF4-*zot*-prophage^+^, T265 and Ba35 (all containing *zot*-encoding prophage, Table 1) were isolated using miniprep and gel extraction kits as previously described [30]. Sequencing of the purified DNA was performed by Illumina HiSeq platform (FIMM Technology Centre, Helsinki, Finland) with paired-end read sizes of 100 bp. Library construction, sequencing and data pipelining were performed in accordance with the manufacturer’s protocols. In order to assemble the replicative form genomes of two novel inoviruses, named pVAI1 (MN200778; coverage 8.6×) and pVAI2 (MN200777; coverage 4.7×), the reads were mapped to previously characterized *zot*-containing contigs of *V. anguillarum* strains Ba35 and T265, respectively, by Geneious software v.10.2.2 (Biomatters Ltd, Auckland, New Zealand) [26,31]. The genome of the replicative form pVAI2 was not fully covered; hence, five sets of primers targeting gap areas were generated and *V. anguillarum* strain T265 was used as a DNA template (Table S1). The PCR products were Sanger sequenced (Table S1) and mapped to the gapped regions in the previously obtained pVAI2 genome using Geneious v.10.2.2 [31]. Thus, the consensus sequence represented the genomic sequence of the replicative forms pVAI2.

### 2.8. Genome Analysis

Pairwise alignment of pVAI1 and pVAI2 using Geneious v.10.2.2 was performed, and potential ORFs were identified using ORF finder [32]. Deduced ORFs were compared with known proteins using the Blastp algorithm against the *Inovirus* (taxid:10861) and the *Inoviridae* (taxid:10860) databases (June 2020). If no significant hits were retrieved using the *Inovirus* and *Inoviridae* databases, the ORFs were searched against the non-redundant database. The best hits for each ORF were reported and the circular sequences were visualized using SnapGene v.4.1.7 (GSL Biotech LLC, San Diego, CA, USA). Whole genome alignments of *V. anguillarum* strains Ba35 and T265 were performed on concatenated genomes using MAUVE [33] in Geneious.

Signal peptides were predicted using SignalP v.4.1 [34]. Phobius [35] and TMHMM v.2.0 [36] were used for the prediction of transmembrane helices. The subcellular localization of the predicted proteins was identified using PSORTb v.3.0.2 [37] and putative promoters were predicted by screening sequences between ORFs of pVAIs for potential σ70 promoters using BPROM [38].

To investigate if the non-coding regions of pVAIs were similar to non-coding sequences of other inoviruses, a Blastn search using the intergenic region 1 (ig-1^pVAI^) and intergenic region 2 (ig-2^pVAI^) as a query against the *Inovirus* (taxid:10861) and *Inoviridae* (taxid: 10860) databases was performed (June 2020). To predict putative replication origins and *attP* sites, sequences from CTXϕ [39,40] were blasted against the genomes of pVAIs and the PF4 *zot*-prophage (Table S2) by Geneious BLAST (v.10.2.2).

In order to evaluate the phylogenetic relationship of the *Inoviridae*-related elements (Table S2), all the conserved ORFs (matching ORF1–8^pVAI^) were concatenated. Each ORF and the concatenated outputs of eight ORFs were aligned by Geneious (v.10.2.2). The phylogenetic trees were constructed using the Neighbor-Joining algorithm (100 bootstrap; Geneious v.10.2.2). For all of the trees, the *C. concisus* strain 13826 prophage CON_phi2 (NC_009802.2) was set as an outgroup. VSK, fs1 and KSF-1ϕ were excluded, due to stop codons in one or more of the ORFs. 

### 2.9. Relative qPCR Analysis of zot Gene Quantities

To isolate DNA for qPCR, the *V. anguillarum* strains PF4*-zot-*prophage^+^, T265 and Ba35 were cultured in triplicates under standard conditions for 24 h. Pelleted cells from the cultures were washed twice in MB, diluted 1:25 in MB and grown under the standard conditions for 24 h. Growth was monitored by measuring OD at 600 nm and at each time point, 1 mL aliquots was heat-treated at 95 °C for 15 min, frozen at −20 °C and used as a template for relative qPCR. In order to estimate the production of virus genomes, the relative gene quantities of *zot* and *dnaJ* were determined by qPCR (Table S1) as previously described [41,42] with an annealing temperature of 58 °C. 

The C_T_ for each reaction was used to quantify *zot* relative to a reference gene, *dnaJ*, using the ΔΔC_T_ method [43], and then normalized to the mean *zot dnaJ*^−1^ ratio of the calibrator isolate, *V. anguillarum* strain PF4*-zot-*prophage^+^. The standard deviation of the ratios was based on the *zot dnaJ*^−1^ ratio for each replicate of the *V. anguillarum* strains T265 and Ba35, normalized to the mean *zot dnaJ*^−1^ ratio of *V. anguillarum* strain PF4*-zot-*prophage^+^.

### 2.10. Host Range Analysis of VAIϕ

To examine if VAIϕ was able to infect other *V. anguillarum* strains, supernatant containing VAI1ϕ was obtained by growing *V. anguillarum* strain Ba35 in standard conditions for 24 h, pelleting cells by centrifugation at 15,000 rpm for 5 min and sterilizing the supernatant by 0.2 µm filtration. Sterility was confirmed by plating 200 µL on 2 plates. Seven *zot*-deficient *V. anguillarum* strains (Table 1; PF4*-zot-*prophage^−^, PF430-3, NB10, 850610-1/6a, 51/82/2, 90-11-286 and HWU53) were then cultured at room temperature with 100 rpm aeration overnight in 1 mL of the sterile VAI1ϕ-containing supernatant. Subsequently, the cultures were serially diluted and plated on MB agar plates and incubated for 24 h. Fifty colonies of each strain were recultured for at least 4 h in standard conditions and used as a template for a duplex PCR assay targeting *zot* and circularized VAI1ϕ elements to examine VAI1ϕ acquisition (Table S1). The colonies containing *zot* and circularized VAI1ϕ gene fragments after phage exposure were selected and assessed for the ability to release VAI1ϕ in the supernatant using PCR targeting *zot* and circularized phage elements as previously described.

### 2.11. Determination of the Integration Site of VAIϕ

To screen the genome sequences of *V. anguillarum* strains T265, Ba35, PF4 and PF430-3 for *dif1* sites, the *V. cholerae dif1* site [39] was compared with the *V. anguillarum* database using Blastn as described earlier (taxid:55601). To investigate if VAIϕ integrated into the *dif1*-like sites in *V. anguillarum*, primer sets were designed so that a primer near the putative attachment site (*attB*) was paired with a primer oriented outward from the boundary of the VAIϕ genome (Table S1). Resulting PCR products of the expected size were interpreted as VAIϕ genome insertions into the potential *attB* sites (Figure 4 and Figure S5; Table S1). For examination of the phage integration site, the two strains that had experimentally acquired the prophage (PF4*-zot-*prophage^−^-VAIϕ and PF430-3-VAIϕ) and the two wild type prophage-containing strains (T265 and Ba35: Table 1) were cultured in standard conditions overnight, along with the prophage-negative *V. anguillarum* strains PF4*-zot-*prophage^−^ and PF430-3 (i.e., no *zot* detection, Table 1). Subsequently, 1 µl of the overnight cultures were used as a template for PCR using a subset of primers and the PCR products were analyzed by gel electrophoresis (Table S1). Then, PCR products of phage genome insertions derived from strains PF4*-zot-*prophage^−^-VAIϕ and Ba35 were purified and the nucleotide sequences were determined by Sanger sequencing (Eurofins, Köln, Germany).

## 3. Results

### 3.1. Clone-Specific Instability of zot-Encoding Prophages Observed in Vibrio anguillarum

Previous reports describe the loss of a *zot*-encoding prophage from *V. anguillarum* strain PF4 upon exposure to a lytic phage, indicating that these prophages are occasionally unstable [27]. PCR-based screening of the stability of *zot*-encoding prophages during growth in *V. anguillarum* revealed that 50 out of 50 colonies of strain T265 and Ba35 contained the *zot* fragment, suggesting that the prophage was preserved in these strains, whereas only 3 out of 50 colonies of strain PF4 retained the *zot* element following culturing, supporting the previous indication of an unstable association of the prophage with strain PF4 [27]. Two PF4-derived clones were selected based on the presence and absence of the *zot* gene, and designated *V. anguillarum* PF4-*zot*-prophage^+^ and PF4-*zot*-prophage^−^, respectively (Table 1). Interestingly, the reassessment of the prophage stability during the 24 h growth of strain PF4*-zot-*prophage^+^, which had retained the prophage during the initial screening experiment, revealed that 50 out of 50 colonies contained the *zot* fragment, suggesting that those clones maintaining the prophage following cultivation established a stable association under the given growth conditions.

### 3.2. Filamentous Phage Particles Were Released in Vibrio anguillarum Strains Ba35 and T265, but Were Not Observed in Strain PF4-zot-Prophage^+^

Assessment of prophage circularization showed that the prophage-containing *V. anguillarum* strains T265 and Ba35 established replicative forms (Figure S1), whereas circularized prophages were not observed in *V. anguillarum* strain PF4-*zot*-prophage^+^, suggesting that the *zot*-encoding prophage was retained in the genome of this strain. Further, the phage *zot*-gene could be PCR amplified from 0.2 µm-filtered and DNase-treated supernatants from strains T265 and Ba35, whereas no amplification of the chromosomal bacterial gene *dnaJ* could be observed (Figure S2). This finding indicated that DNase-resistant *zot*, and thus *zot*-encoding phages, were present in the supernatants of strains T265 and Ba35 (Figure S2). Contrarily, no *zot* amplification was detected in the supernatant of strain PF4-*zot*-prophage^+^, demonstrating that this strain did not spontaneously produce phage (Figure S2), but rather contained a stable, non-inducible, *zot*-encoding prophage. 

**Figure 1:**
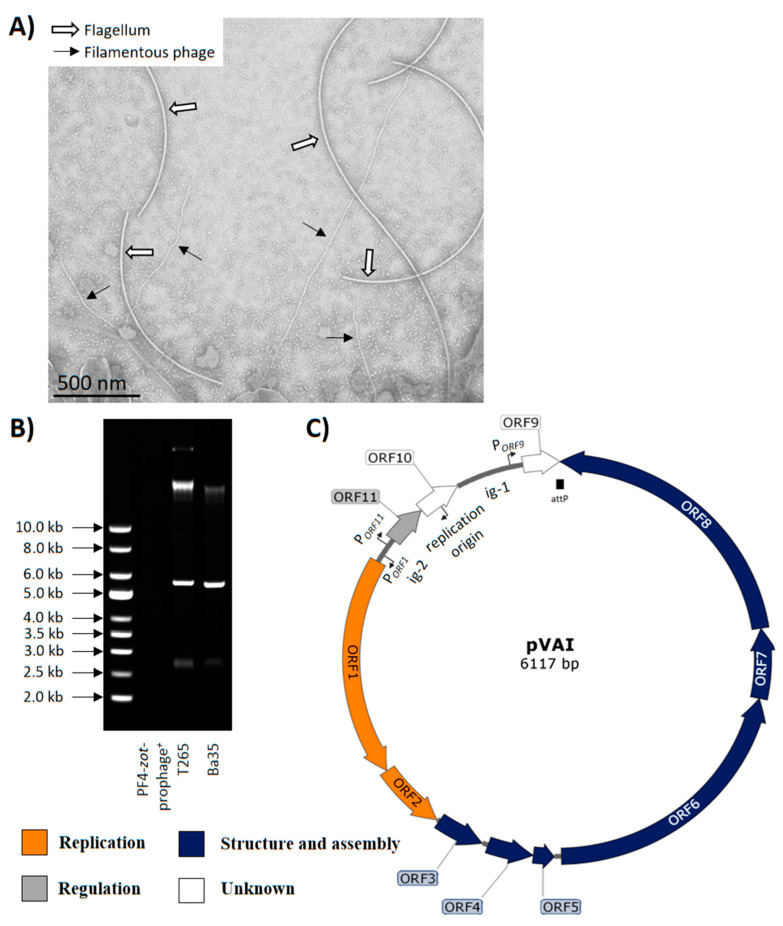
Electron micrograph of VAIϕ particles and schematic representation of pVAIs. (**A**) Transmission electron micrographs of overnight cultures of *Vibrio anguillarum* strain Ba35. (**B**) Extraction of replicative forms in *V. anguillarum* strains separated on a 0.8% agarose gel. A supercoiled DNA ladder is shown to the left. (**C**) Schematic representation of pVAIs, the predicted *attP*, replication origin, promoters, intergenic regions and ORF organization. ORFs are represented by arrows oriented in the direction of transcription. The colors were assigned according to the possible role of each ORF as shown in the figure. The *attP* is indicated as a black rectangle and the replication origin and promoters are represented by bent arrows pointing in the direction of replication and transcription, respectively.

Previously characterized inoviruses exhibited a buoyant density of ~1.3 g mL^−1^ in CsCl [49,50]. Examination of CsCl gradients loaded with 0.2 µm-filtrates of mitomycin C induced supernatants of *V. anguillarum* strains PF4 and T265 and subsequent PCR analysis did not reveal any amplification of the *zot* gene in PF4, but for T265, the *zot* amplification was most intense, at a buoyant density of 1.30–1.34 g mL^−1^ (Figure S3). Furthermore, electron micrographs of a *V. anguillarum* strain Ba35 overnight culture revealed 7 nm wide filamentous particles (Figure 1A). These filaments were not observed in strain PF4-*zot*-prophage^−^, which does not contain a *zot*-encoding prophage. Collectively, these results suggested that the *zot*-encoding prophages established circularized elements and were released as filamentous particles in the supernatant of the strains Ba35 and T265. The filamentous virions obtained from *V. anguillarum* strains Ba35 and T265 were termed VAI1ϕ and VAI2ϕ, respectively. Similarly, the replicative forms from strains Ba35 and T265 were designated pVAI1 and pVAI2, respectively. Collectively, these were referred to as VAIϕ and pVAIs. 

### 3.3. Characterization and Annotation of pVAIs in Vibrio anguillarum

In order to determine the genomic sequences of pVAIs, replicative forms were extracted, revealing the presence of one distinct DNA band in each of the *V. anguillarum* strains T265 and Ba35 (Figure 1B). Contrarily, DNA bands were not detected in *V. anguillarum* strain PF4-*zot*-prophage^+^ (Figure 1B), emphasizing that the *zot*-encoding prophage in strain PF4-*zot*-prophage^+^ was not replicated as an extrachromosomal element. Sequencing of the putative replicative forms revealed two novel *Inovirus* genomes, pVAI1 and pVAI2. The genomes of pVAIs consisted of 6117 bp, were predicted to encode 11 ORFs, had a GC content of 42.5% and contained two intergenic regions corresponding to conserved sequences of inoviruses [13,28]. Global alignment of the genomes of pVAI1 and pVAI2 exhibited a pairwise identity of 99.95%. The *V. anguillarum* strains Ba35 and T265 exhibited 94.20% nucleotide identity at the genome level. These findings suggested that almost identical inoviruses were present in the closely-related strains Ba35 and T265; thus, we assume that the data regarding VAI1ϕ and VAI2ϕ can be extrapolated to represent VAIϕ inoviruses in general in these strains.

**Figure 2:**
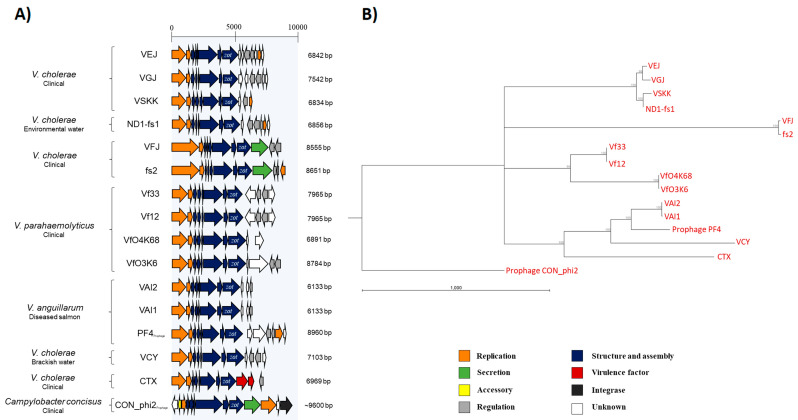
Schematic representation of annotated prophages, inoviruses and their phylogenetic relationship based on conserved genes. (**A**) Inoviruses and prophages in *Vibrio* hosts and their source of isolation; a *Campylobacter concisus* prophage as the outgroup was included. Linearized *Inovirus-*related sequences were aligned for visual comparison and arrows represent the ORFs and their transcription direction. Colors were assigned according to the putative function, which was predicted based on the ORF synteny, size, presence of transmembrane helices, signal peptides and their predicted subcellular localization. (**B**) Phylogenetic relationship of *Inoviridae-*related vibriophages and a prophage based on concatenated amino acid sequences of 8 conserved ORFs involved in replication, structure and assembly. The concatenated sequence was used to construct the phylogenetic tree using the maximum likelihood algorithm (100 bootstraps). Only bootstrap values supporting branches > 80 are shown. VSK, KSF-1ϕ and fs1 were excluded of this analysis as mutations caused premature stop codons in some of the generic ORFs.

Filamentous vibriophages can be annotated into functional modules based on gene sequence, size and synteny [4,13]. This annotation system was used to predict the role of the pVAIs’ ORFs (Figure 1C; Table S3). The annotations of the novel inoviruses VAI1ϕ, VAI2ϕ, the *V. anguillarum zot*-encoding prophages and publically available filamentous vibriophage genomes (Table S2; excluding VSK, KSF-1ϕ and fs1) revealed an overall similar gene synteny divided in replication, structure and assembly modules (Figure 2A; Table S3). Specifically, ORF1^pVAI^ and ORF2^pVAI^ had comparable length, synteny and 44%–75% amino acid identity to replication modules of previously characterized filamentous vibriophages (Figure 1C and Figure 2A; Table S3). ORF3^pVAI^ to ORF8^pVAI^ showed 27%–73% amino acid identity, similar size and synteny to structural and assembly proteins in other filamentous vibriophages (Figure 1C and Figure 2A; Table S3) [30,32,51]. Interestingly, ORF8^pVAI^ protein was similar to the *zot*-like proteins of the described filamentous phages VCYϕ, KSF-1ϕ, VfO3K6, Pf1 and CTXϕ, where the protein is involved in the assembly and secretion of the virus and reported as a putative toxin in the latter (Tables S3 and S4) [13,14]. ORF9^pVAI^ and ORF10^pVAI^ proteins showed 74%–95% amino acid identity to hypothetical bacterial proteins (Table S3). ORF11^pVAI^ gene product displayed 95% amino acid identity and similar size and synteny compared to the RstR protein of CTXϕ (ACV95544.1), which serves as a transcriptional repressor of the phage genes (Figure 1C and Figure 2A; Table S3) [52]. 

Like the majority of filamentous vibriophages, a gene encoding a secretin responsible for virion extrusion could not be identified in VAIϕ and the prophage of *V. anguillarum* strain PF4 (Figure 2A). Thus, VAIϕ likely utilizes a host-encoded secretin for extrusion, as has been shown for the filamentous vibriophage CTXϕ [53,54]. Blastn analysis revealed a putative origin of replication, a single *attP* and intergenic regions containing putative σ70 promoters related to previously described filamentous vibriophages (Figure 1C). 

### 3.4. Vibrio anguillarum Inoviridae-Related Elements Are Closely Related to Vibrio cholerae Inoviruses

To examine the phylogenic relationship among *V. anguillarum Inovirus*-related elements and characterized filamentous vibriophages, the amino acid sequences of the generic ORFs were concatenated and compared in a phylogenetic tree, using an *Inoviridae*-related prophage from *C. concisus* as the outgroup (Figure 2). According to this phylogenetic tree, the *zot*-prophage of *V. anguillarum* strain PF4 and pVAIs were closely related to the environmentally and clinically isolated *V. cholerae* phages VCYϕ and CTXϕ (Figure 2). Furthermore, a group of the *V. anguillarum Inoviridae*-related elements, VCYϕ and CTXϕ, clustered separately from other *V. cholerae* (VEJϕ, VGJϕ, VSKK, ND1-fs1, VFJϕ and fs2) and *V. parahaemolyticus* (Vf33, Vf12, VfO4K68 and VfO3K6) inoviruses (Figure 2B).

*V. cholerae* filamentous phage genomes are highly mosaic and probably assembled through horizontal gene transfer [18,30,51]. To assess the genomic structure of VAIϕ and the *Inoviridae*-related prophage in *V. anguillarum* strain PF4, separate phylogenetic trees were constructed for each conserved ORF and the relatedness was summarized (Figure S4). Expectedly, the structure-and-assembly ORFs of VAIϕ and the PF4 prophage were related to the *V. cholerae* phage VCYϕ (Figure S4). However, the replication ORFs of VAIϕ and the PF4 prophage were related to CTXϕ and to a group of *V. parahaemolyticus* filamentous phages, respectively (Figure S4). Additionally, ig-1^pVAI^ and ig-2^pVAI^ exhibit 68% and 87% DNA identity to *V. parahaemolyticus* phages and CTXϕ, respectively. Also, the *zot*-encoding prophage in *V. anguillarum* strain PF4 contained a 238 bp ig-2-like region adjacent to the *zot*-like gene that exhibited 67.87% nucleotide identity to the *V. parahaemolyticus* phages VfO3K6 and VfO4K68. Collectively, VAIϕ and the *Inoviridae*-related prophage in *V. anguillarum* strain PF4 exhibited mosaic genomes with modules and intergenic regions related to diverse *V. cholerae* and *V. parahaemolyticus* filamentous phages.

### 3.5. VAIϕ Reached High Estimated Genome Copies mL^−1^ In Vitro

**Figure 3:**
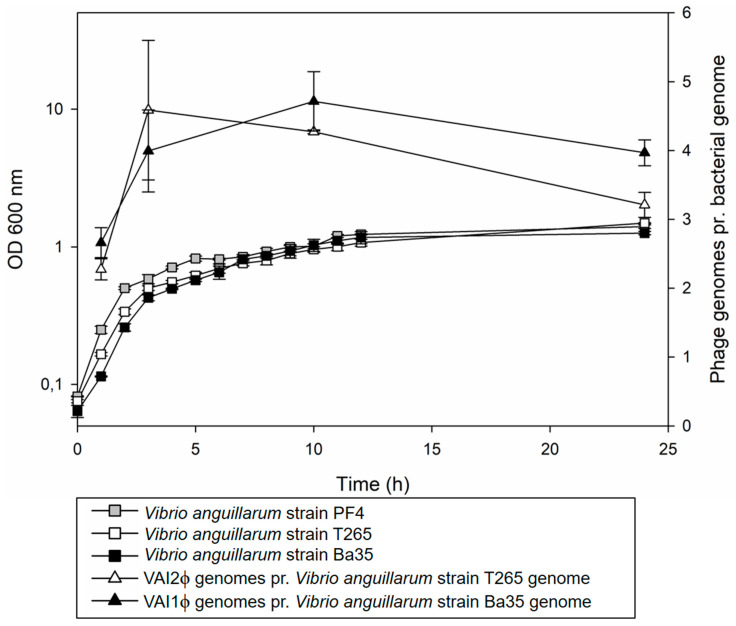
Bacteria growth and replication of phage genomes. The relative amount of *zot* and *dnaJ* was quantified by qPCR in cultures of *Vibrio anguillarum* strains. The *zot dnaJ*^−1^ ratios in T265 and Ba35 were normalized to the measures of *V. anguillarum* strain PF4-*zot*-prophage^+^, which appears to contain a stable non-inducible *zot*-encoding prophage. After normalizing to PF4-*zot*-prophage^+^, the relative gene quantities in T265 and Ba35 can be interpreted as an estimate of phage genomes (*zot*) pr. bacterial genome (*dnaJ*). Error bars indicate the standard deviation of biological triplicates.

Enumeration of filamentous vibriophages has often been done after 0.22 µm filtration of supernatants [13,51], which may considerably underestimate the number of induced phages, due to retention of phage filaments on the filter. Hence, in this study, a filtration-independent qPCR approach was applied to estimate the phage genome copies in *V. anguillarum* hosts by quantifying a phage-related gene (*zot*) and a chromosomal reference gene (*dnaJ*) relative to the calibrator strain PF4-*zot*-prophage^+^, which does not produce replicative forms and therefore is expected to only carry one copy of each of the *zot* and *dnaJ* genes per bacterial genome (Table S1). Thus, by normalizing the *zot dna^J^*^−1^ ratio in *V. anguillarum* strains T265 and Ba35 to the quantification in strain PF4-*zot*-prophage^+^, the values may be interpreted as a quantification of phage genomes per bacterial genome (Figure 3). 

The result showed that in the early exponential growth phase, from 1 h to 3 h of cultivation, the *zot dnaJ*^−1^ ratio increased from 2.27 and 2.66 to 4.59 and 3.99 in strains T265 and Ba35, respectively (Figure 3). At the stationary growth phase, after 3 to 10 h of cultivation, the ratio stabilized between 4 and 5, followed by a decrease to 3.21 and 3.97 *zot dnaJ*^−1^ after 24 h incubation of strains T265 and Ba35, respectively (Figure 3). Thus, under these conditions VAIϕ was spontaneously induced and replicated during host exponential growth rising to ~4 phage genomes per bacterial genome at the host stationary growth phase (Figure 3). As stationary phase cultures of *V. anguillarum* typically comprise ~4 × 10^9^ CFU mL^−1^ under in vitro conditions, the data suggest a conservative estimate of VAIϕ abundance of approximately 10^10^ phage genomes mL^−1^ at the host stationary growth phase.

### 3.6. VAIϕ Can Disseminate and Propagate in Vibrio anguillarum

To evaluate whether VAIϕ was able to disseminate among *V. anguillarum* strains, 0.2 µm-filtered supernatant containing VAI1ϕ was exposed to selected prophage-negative *V. anguillarum* strains, and screened for phage acquisition (Table 1 and S1). Based on PCR analysis, 1 out of 50 *V. anguillarum* strain PF4-*zot*-prophage^−^ and 1 out of 100 *V. anguillarum* strain PF430-3 colonies were infected after overnight cultivation with VAI1ϕ (Table S1), whereas no infection was observed in the *V. anguillarum* strains NB10, 850610-1/6a, 51/82/2, 90-11-286 and HWU53 among 50 colonies examined. The analysis also showed that VAI1ϕ existed as circular elements in the newly-infected *V. anguillarum* strains PF4-*zot*-prophage^−^ and PF430-3, and subsequent recultivation of the infected clones revealed the presence of nuclease-resistant phage DNA and circularized phage elements in 0.2 µm-filtered supernatant of these cells (Table S1). This result suggested that the newly infected isolates, termed *V. anguillarum* strain PF4-*zot*-prophage^−^-VAIϕ and PF430-3-VAIϕ, produced VAI1ϕ particles.

### 3.7. VAIϕ Can Integrate at Several Sites in Both Vibrio anguillarum Chromosomes

**Figure 4:**
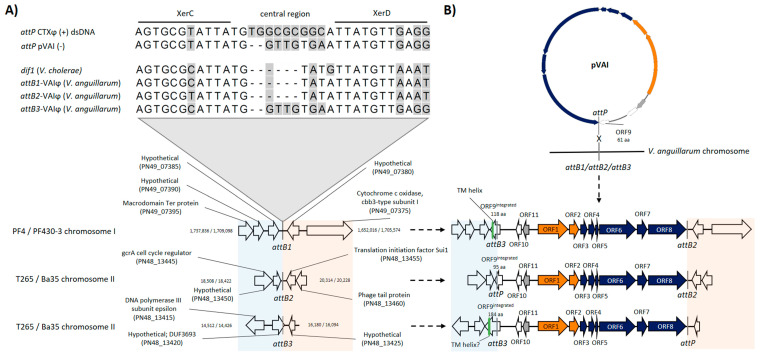
pVAIs *attP* and insertion of VAIϕ genome in *Vibrio anguillarum* chromosomes. (**A**) Alignment of CTXϕ and pVAIs putative *attP* plus *V. cholerae dif1* [39] and *V. anguillarum dif1*-like (*attB*) sites and their genomic positions as indicated by a gray line in chromosomes of *V. anguillarum* hosts where a VAIϕ genome insertion was observed. The extended lines indicate XerC and XerD binding sites and are separated by a central region. Vertical lines indicate alignment gaps and non-identical nucleotides are shaded. (**B**) The fate of VAIϕ genome insertions in *V. anguillarum*. The assumed strand exchange altered the *att* sites upon VAIϕ genome insertion into *attB1* in PF4-*zot*-prophage^−^-VAIϕ and resembled *attB* sites observed in *V. anguillarum* strains Ba35 and T265. The presumed strand exchange conserved the observed *attP* and *attB* sites following VAIϕ genome insertion into *attB2* and *attB3* in strains Ba35 and T265. Open arrows represent ORFs oriented in the transcription direction and the color indicate the putative functions of structure-and-assembly, replication, regulation and other/hypothetical for blue, orange, gray and white, respectively. The green rectangles in (B) represent the presence of predicted transmembrane domains in ORF9^intergrated^ (Table S5).

Genome sequencing of the prophage-bearing strains did not reveal the integration site of VAIϕ prophage as the automated genome assembly sorted the prophage genome as a contig that did not overlap other contigs of the bacterial genome. Therefore, it was necessary to confirm that VAIϕ can indeed insert its chromosome into the bacterial genome. Studies of CTXϕ showed that *dif1* is the integration site in *V. cholerae* [39] and a search for this specific region in *V. anguillarum* revealed *dif1*-like sites located in both chromosome I and II (Figure 4A). Subsequent PCR analysis with flanking primers confirmed five intact *attB* sites out of the six predicted sites (Figure S5). Specifically, the VAIϕ genome was inserted into the predicted *attB* site in chromosome I of *V. anguillarum* strains PF4-*zot*-prophage^−^-VAIϕ and PF430-3-VAIϕ and into two predicted *attB* sites only separated by ~4000 bp in chromosome II of *V. anguillarum* strains T265 and Ba35 (Figure 4B). The results suggested that VAIϕ integrated its genome in a chromosome-unspecific manner into the *dif1*-like (*attB*) sites in *V. anguillarum* (Figure 4). Additionally, PCR amplification of fragments corresponding to both VAIϕ genome insertions and excisions in the bacterial chromosomes were detected for the phage bearing strains (Figure S5), indicating that excision and integration of the VAIϕ genome in *V. anguillarum* was dynamic. The two confirmed *dif1*-like sites (positive PCR signals with chromosome-targeted primers) where VAIϕ genome insertion was not observed were identical and contained two nucleotide changes at conserved positions in the central region, possibly hindering VAIϕ genome insertion into this site (Figure S6).

Sanger sequencing of phage genome insertion sites (Table S1) revealed that while the dynamic excision and integration preserved the bacterial ORFs intact and maintained the reading frame of the phage gene ORF9^pVAI^, the sequence of ORF9^pVAI^ was extended. The extended form of the ORF could potentially have an altered function, as it contained a predicted transmembrane domain in the C-terminal end when the phage genome was inserted into *attB1* in chromosome I (i.e., strains PF4-*zot*-prophage^−^-VAIϕ and PF430-3-VAIϕ) and into *attB3* in chromosome II (i.e., strains Ba35 and T265) (Figure 4B and Table S5). Moreover, the results revealed that after integration in strain PF4-*zot*-prophage^−^-VAIϕ, the phage *attP* and bacterial *attB1* sites had presumably exchanged strands and resembled the *attB2* and *attB3* sites originally observed in strains Ba35 and T265 (Figure 4B). However, the *attP* and *attB* sites in strain Ba35 remained intact after phage integration.

## 4. Discussion

The mosaic genome structure of the *zot*-encoding VAIϕ filamentous phages was closely related to that of the *V. cholerae* phages VCYϕ and CTXϕ (Figure 2 and Figure S4). Prophage-encoded *zot*-like genes are linked with various human pathogens [14,19,23,55] and in *V. cholerae*, Zot peptides have been proposed to act as toxins relying on a specific domain (FCIGRL) that, upon binding to a receptor, results in tight junction disassembly in the small intestine of mammals [56,57]. In VAIϕ and other filamentous vibriophages, the Zot-like proteins do not contain this specific domain found in *V. cholerae* [56,57]; thus, it seems unlikely that these proteins would have a similar toxic effect on the small intestines of mammals. However, lysogenic conversion by *zot*-encoding *Inoviridae-*related prophages has also been proposed as a virulence mechanism in the coral pathogen *V. corallilyticus* [18,58], and the fish pathogens *V. harveyi* [59] and *V. anguillarum* [23,27], suggesting that integration of VAIϕ may contribute to the virulence properties of the lysogenized host. Recently, a large-scale functional analysis of virulence genes in phage genomes associated with coral reefs showed that pathogenic functions encoded by phages enable bacteria to recognize and invade animals, and suggested that lysogenic conversion is a widespread virulence mechanism among marine pathogens [60]. Furthermore, high abundance of *Inoviridae*-related reads in a metavirome comparison among animal-associated habitats supports that filamentous (pro)phages or their gene products could be involved in bacterial adaptation to their animal host environment [44]. 

VAIϕ was spontaneously produced by specific *V. anguillarum* lysogens and was capable of cross-infection and site-specific integration into non-lysogenic *V. anguillarum* host genomes (Figure 4). The observed dynamics of release, acquisition and subsequent loss of *Inoviridae*-elements in *V. anguillarum* [27] further suggested that these sequences are subject to a rapid gene flux, thus possibly playing a role in *Vibrio* adaptation to changing environmental conditions [30]. Collectively, these data contribute to the emerging perception of *zot*-encoding inoviruses as important drivers of host functional properties beyond virulence in human *Vibrio* pathogens [18,23].

The mosaic genome structure of pVAIs and related phages supports the notion that horizontal gene transfer is a key factor in the evolution of filamentous phages because it allows the assembly of highly mosaic genomes, possibly by co-infection, and subsequent recombination of the genomes (Figure S4). Combined with the observed distribution of highly conserved core prophage genomes across *Vibrio* species [3,23], this theory suggests that interspecies evolution by prophage exchange may occur continuously in *Vibrio* populations [30,51]. 

VAIϕ establishes lysogeny at several chromosomal sites (Figure 4) and our results indicate the simultaneous presence of integrated and excised VAIϕ genomes in *V. anguillarum* populations (Figure S5), suggesting a dynamic regulation of the states of these elements. Several filamentous vibriophages are inducible by mitomycin C, while the majority, as VAIϕ, are spontaneously replicated and released under standard laboratory conditions (Figure 3, Figures S1 and S2) [13,30,51]. However, the observed differences in the stability of the *zot-*prophage-host interaction between *V. anguillarum* strains indicate that multiple factors are involved in the regulation of induction, replication and dissemination of these prophages. This corresponds to observations in *V. cholerae* that CTXϕ is disseminated whereas VGJϕ is integrated during in vivo cultivation [13,61] and that the distribution of replicative forms and prophages of the vibriophage VCYϕ depends upon environmental conditions [30]. Thus, unknown environmental or cell density related factors likely influence the integration, induction and production of these specific prophages [8,30,61]. 

The attachment site of pVAIs is located inside ORF9^pVAI^ and the integration event prolonged ORF9^pVAI^ in the examined *V. anguillarum* strains, possibly altering its putative function (Figure 1C and Figure 4, Table S5). Furthermore, modification of the apparent integration hotspots in the *V. anguillarum* strains PF4-*zot*-prophage^−^-VAIϕ and PF430-3-VAIϕ (Figure 4B), by dynamic integration and excision of the phage genomes, potentially changes the affinity for integration of mobile genetic elements, as it has been shown for the satellite phage RS1 in *V. cholerae* [62]. Thus, VAIϕ infection may alter the host phenotype beyond the evident provision of phage-encoded ORFs. 

Together with recent reports of widespread distribution of filamentous phages [3], particularly in vibrios [23], the current study has thus emphasized the need for improving our understanding of the potential of inoviruses as drivers of phenotypic diversification and transmitters of virulence genes in human and animal pathogens. While this study confirmed the integration of VAIϕ genomes, a relative distribution of the different states of the phage genome was not determined, and more studies are required to explore the distribution and phenotypic implications of the different states of VAIϕ in the host. Furthermore, recent indications that prophages are implicated in the rise of pathogens in disturbed marine ecosystems [60] underline the importance of understanding the environmental cues regulating the phage states. Particularly, the role of temperature for *Inovirus* dynamics is important to examine, since the global temperature increase has shown to favor *Vibrio* propagation in the oceans [63]. Thus, it will be important to unravel how environmental perturbations (e.g., increasing temperature and eutrophication) affect *Inovirus* behavior, dissemination and implications for host phenotype. 

## Figures and Tables

**Table 1:** Overview of the *Vibrio anguillarum* strains used in this study, their source of isolation and the detection of *zot*-encoding prophages.

*Vibrio anguillarum* Strains	Isolated From	*zot*-Encoding Prophage Detected	Virulence [44] *	Reference
PF4	Atlantic salmon, Chile	+	High	[45]
PF4-*zot*-prophage^+^	Derivative of PF4	+	^−^	This study
PF4-*zot*-prophage^−^	Derivative of PF4	^−^	^−^	This study
PF4-*zot*-prophage^−^-VAIϕ	PF4-*zot*-prophage^−^ infected with VAI1ϕ	+	^−^	This study
PF430-3	Derivative of PF4	^−^	High	[46]
PF430-3-VAIϕ	PF430-3 infected with VAI1ϕ	+	^−^	This study
T265	Atlantic salmon, UK	+	Low	[29]
Ba35	Sockeye salmon, USA	+	Low	[29]
NB10	Rainbow trout, Sweden	^−^	Low	[47]
850610-1/6a	Rainbow trout, Denmark	^−^	Low	[29]
51/82/2	Rainbow trout, Germany	^−^	Low	[29]
90-11-286	Rainbow trout, Denmark	^−^	High	[48]
HWU53	Rainbow trout, Denmark	^−^	Medium	[29]

* The virulence ranking is based on a previous study that examined the virulence of *V. anguillarum* strains toward fish larvae.

## Supplementary Materials

The following are available online at https://www.mdpi.com/1999-4915/12/7/730/s1, Figure S1: Evaluation of circularization of *zot*-containing prophages in *Vibrio anguillarum* strains. Figure S2: Assessment of nuclease-resistant *zot* genes in supernatants of *Vibrio anguillarum* strains. Figure S3: PCR amplification of *zot* accumulated at 1.3–1.34 g mL^−1^ in *Vibrio anguillarum* strain T265. Figure S4: Mosaic genome structures among conserved genes in filamentous vibriophages. Figure S5: Schematic overview of the PCR analysis of VAIϕ genome insertions into *dif1*-like sites. Figure S6: Alignment of phage and bacterial attachment sites with a *dif1*-like site. Table S1: List of primer sequences and their purpose in this study. Table S2: List of phages and prophage host sequences and their respective accession no. used in this study [64,65,66,67,68,69,70,71,72,73]. Table S3: Annotation of pVAIs’ ORFs. Table S4: ORF8 of pVAIs searched against the *Inovirus* (taxid: 10861) and *Inoviridae* (taxid: 10860) databases. Table S5: Annotation of ORF9 before and after integration in *Vibrio anguillarum* genomes.
