# Supplementary material for: Beyond Cholera: Characterization of zot-Encoding Filamentous Phages in the Marine Fish Pathogen Vibrio anguillarum
Source: Viruses. 2020 Jul 6;12(7):730. doi: 10.3390/v12070730 (PMC7412436; doi:10.3390/v12070730)
Supplement: Supplementary file 1 [file viruses-12-00730-s001.pdf]

Beyond cholera: characterization of *zot*-encoding filamentous phages in the marine fish pathogen *Vibrio anguillarum*

Jesper Juel Mauritzen[a], Daniel Castillo[a], Demeng Tan[b], Sine Lo Svenningsen[b] & Mathias Middelboe[a#]

a) Marine Biological Section, University of Copenhagen, Strandpromenaden 5, DK-3000, Helsingør, Denmark.

b) Section for Biomolecular Sciences, University of Copenhagen, Ole Maaløes Vej 5, DK-2200, København N, Denmark

Supplemental Material

A)

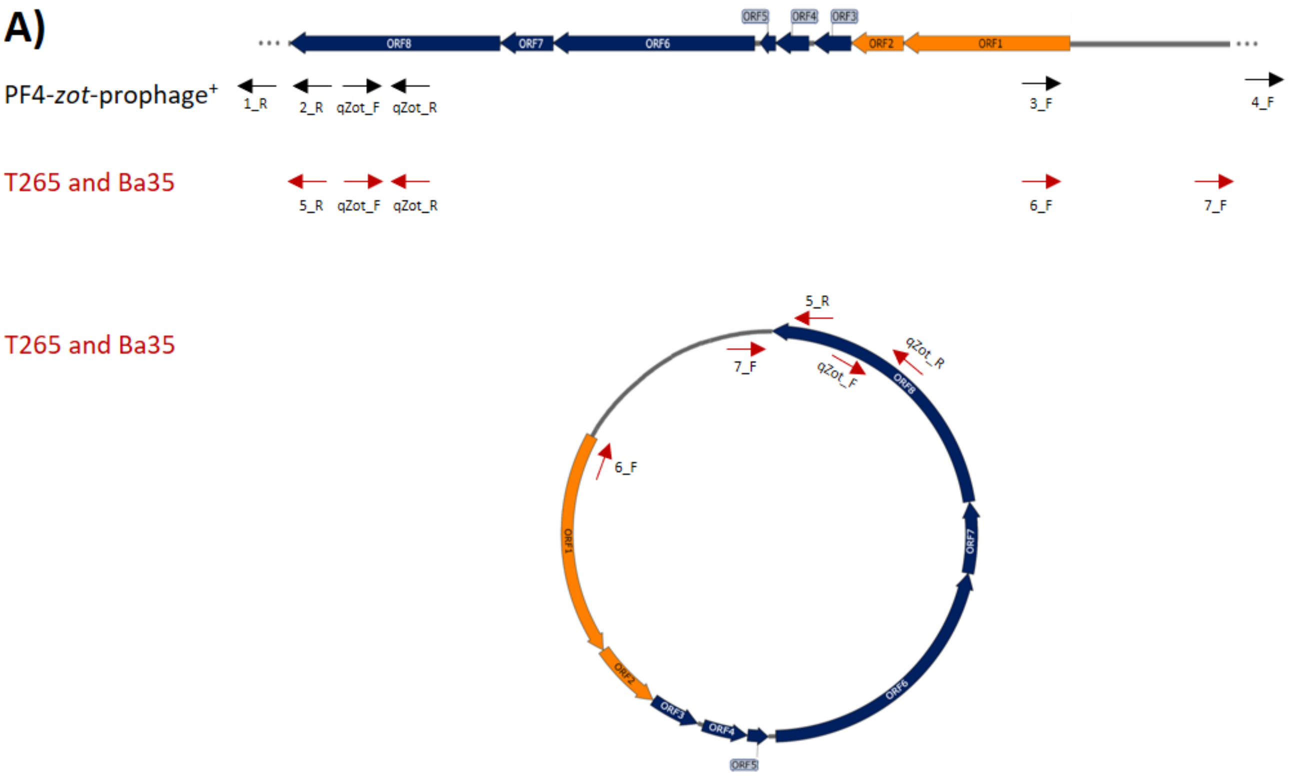
ORF8
ORF7
ORF6
ORF5
ORF4
ORF3
ORF2
ORF1
PF4-zot-prophage+
1_R
2_R
qZot_F
qZot_R
3_F
4_F
T265 and Ba35
5_R
qZot_F
qZot_R
6_F
7_F
T265 and Ba35
5_R
7_F
qZot_R
qZot_F
6_F

B)

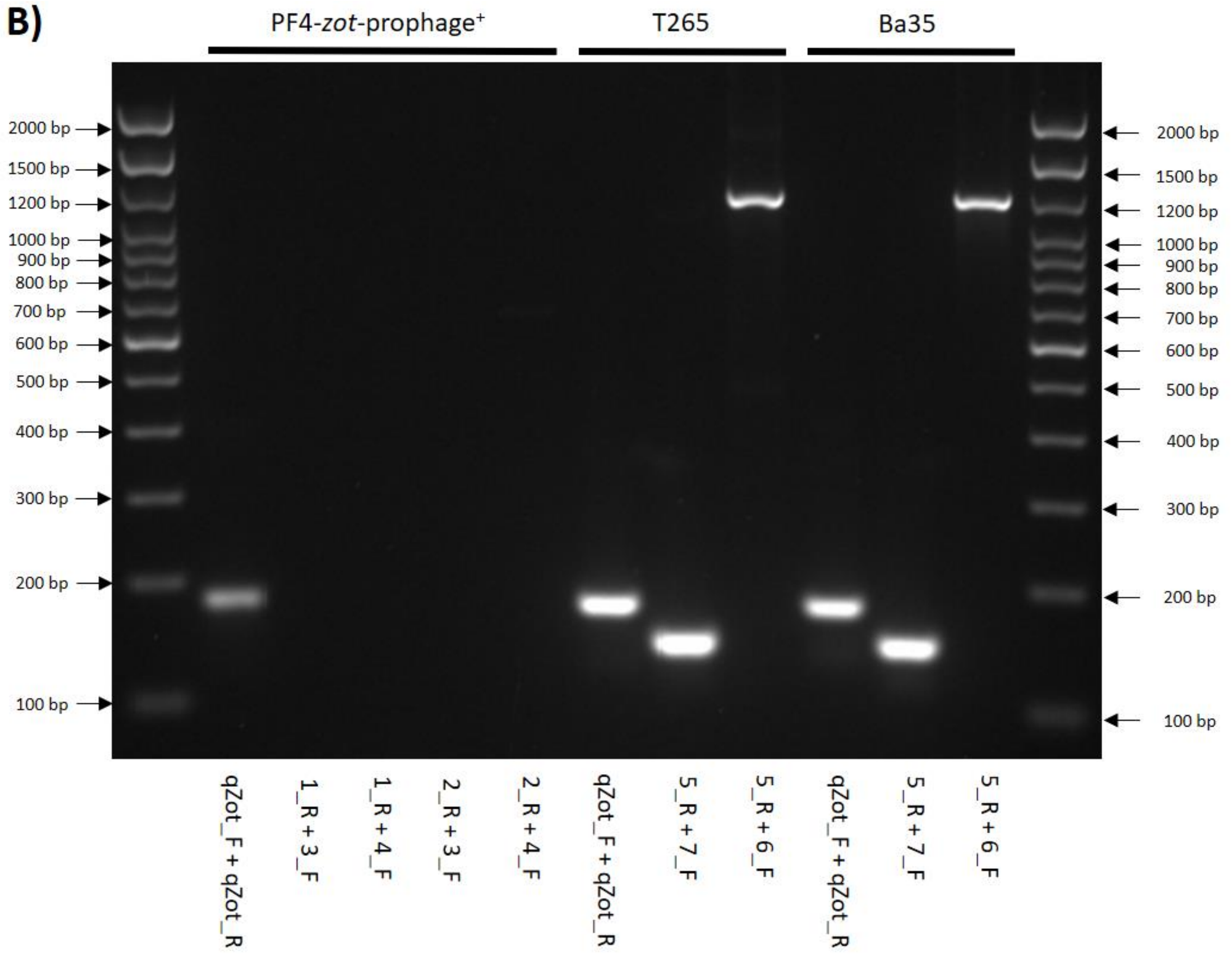
PF4-zot-prophage+
T265
Ba35
2000 bp
1500 bp
1200 bp
1000 bp
900 bp
800 bp
700 bp
600 bp
500 bp
400 bp
300 bp
200 bp
100 bp
qZot_F + qZot_R
1_R + 3_F
1_R + 4_F
2_R + 3_F
2_R + 4_F
qZot_F + qZot_R
5_R + 7_F
5_R + 6_F
qZot_F + qZot_R
5_R + 7_F
5_R + 6_F

Figure S1. Evaluation of circularization of *zot*-containing prophages in *Vibrio anguillarum* strains. Primers targeting *zot* and primers directed at the boundaries of the prophage regions were used in a PCR assay to evaluate the presence of circularized elements in overnight cultures of the *V. anguillarum* strains PF4-*zot*-prophage$^+$, T265 and Ba35 (Supplementary Table S2). **A**) Shows the position of the primers on the linear and circularized version of the phage genome. **B**) The PCR products were separated on a 2 % agarose gel by electrophoresis. The two primers used for each PCR reaction are indicated below the gel. Linear DNA ladders are indicated in the outer lanes. The expected product size of qZot_F + qZot_R is 180 bp. The results demonstrate the presence of circularized prophage elements in strains T265 and Ba35 but not in PF4-*zot*-prophage$^+$.

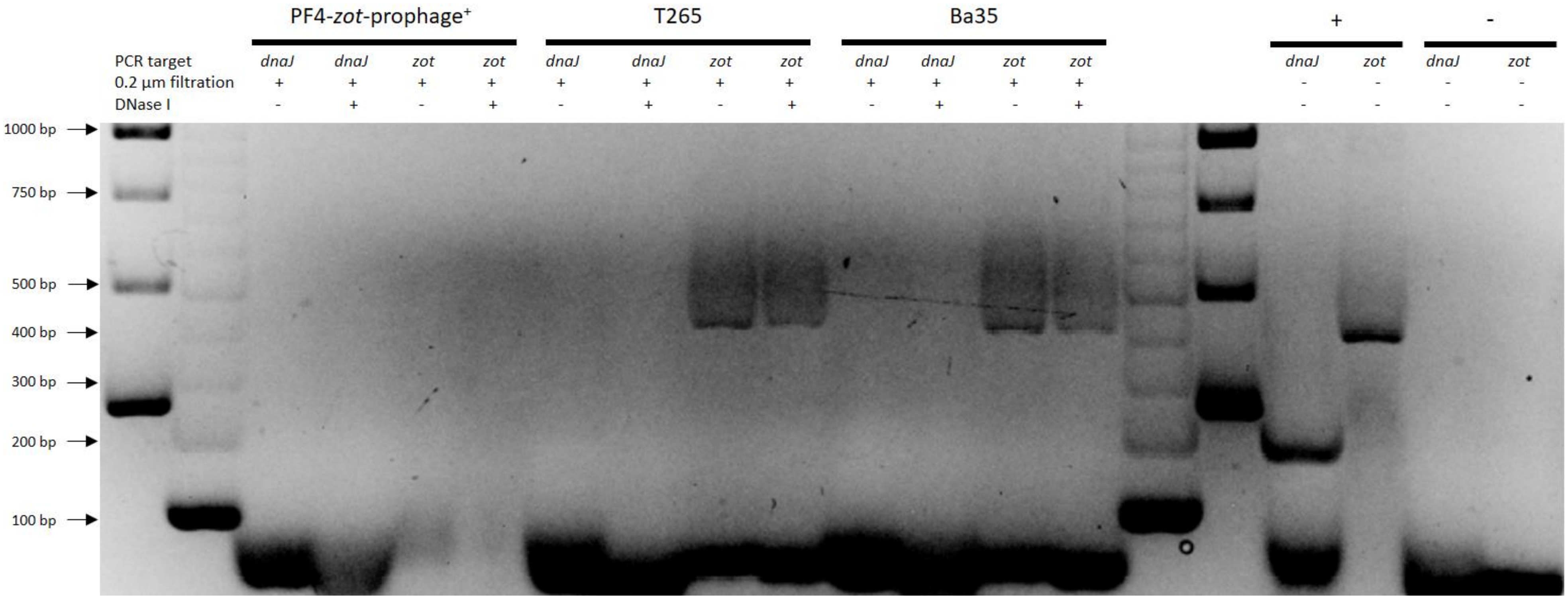

Figure S2. Assessment of nuclease-resistant *zot* genes in supernatants of *Vibrio anguillarum* strains. Supernatants of overnight cultures were 0.2 µm-filtered and treated +/- DNase I. Subsequently, DNase I was inactivated and the samples were used as template for PCR targeting either *zot* or *dnaJ* (Table S2). The PCR products were separated on a 2 % agarose gel by electrophoresis. Two ladders on the left are by 250 and 100 bp steps, respectively. Positive controls (+) and negative controls (-) were generated using non-filtered PF4-*zot*-prophage$^+$ cultures and MB as templates, respectively. A band at position 406 bp indicates the presence of the *zot* gene in the *zot* PCR reactions, and a band at position 191 bp indicates the presence of the *dnaJ* gene in the *dnaJ* PCR reactions. The results demonstrate the presence of free phage particles in supernatants from strains T265 and Ba35 but not from PF4-*zot*-prophage$^+$.

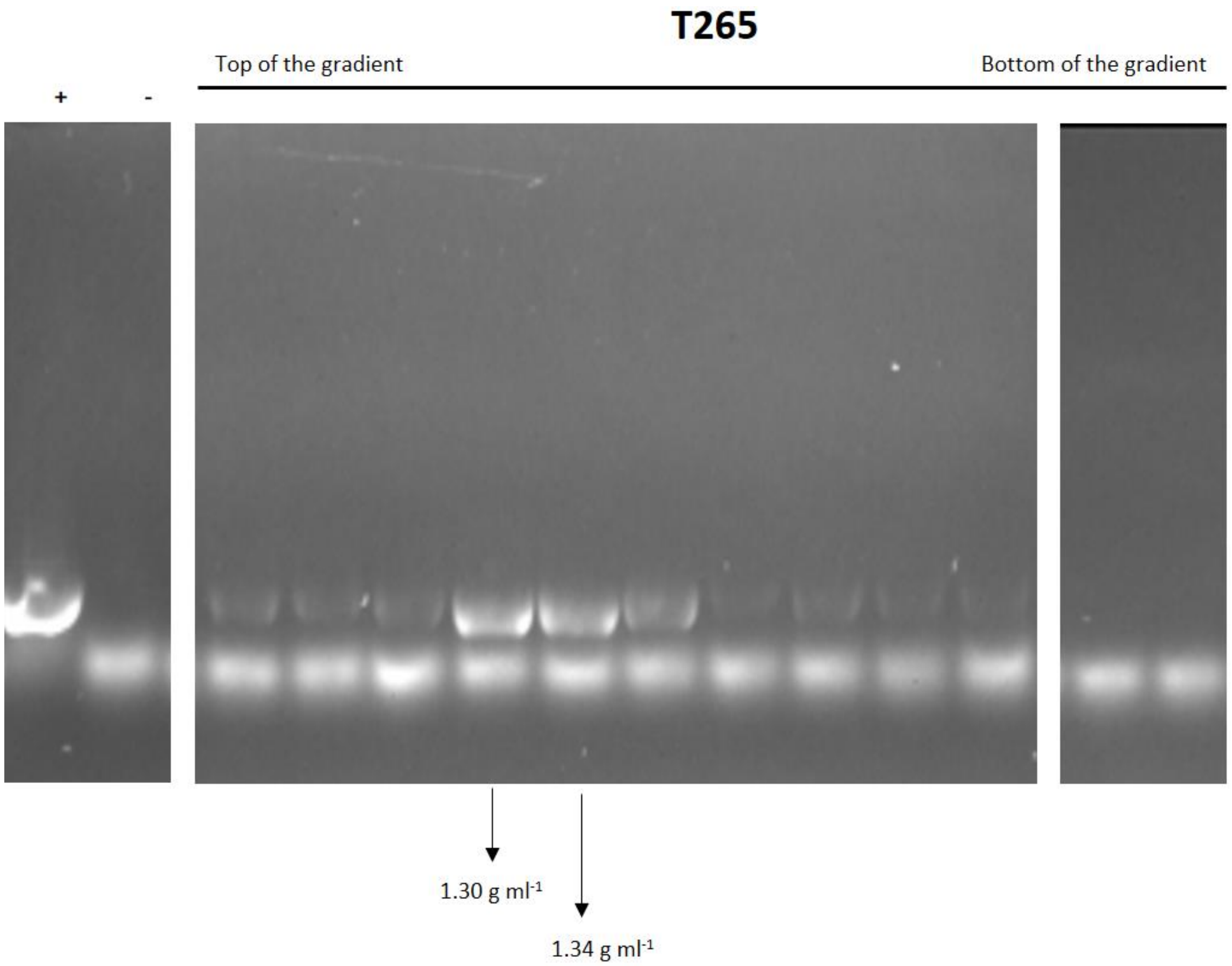

Figure S3. PCR amplification of *zot* accumulated at 1.3-1.34 g ml^{-1} in *Vibrio anguillarum* strain T265. PCR amplification of *zot* in supernatants of *V. anguillarum* strain T265 separated on cesium chloride gradients suggested that the *zot* genes accumulated at a buoyant density of 1.3-1.34 g ml^{-1}. Positive control (+) and negative control (-) are non-filtered *V. anguillarum* strain T265 and MB, respectively.

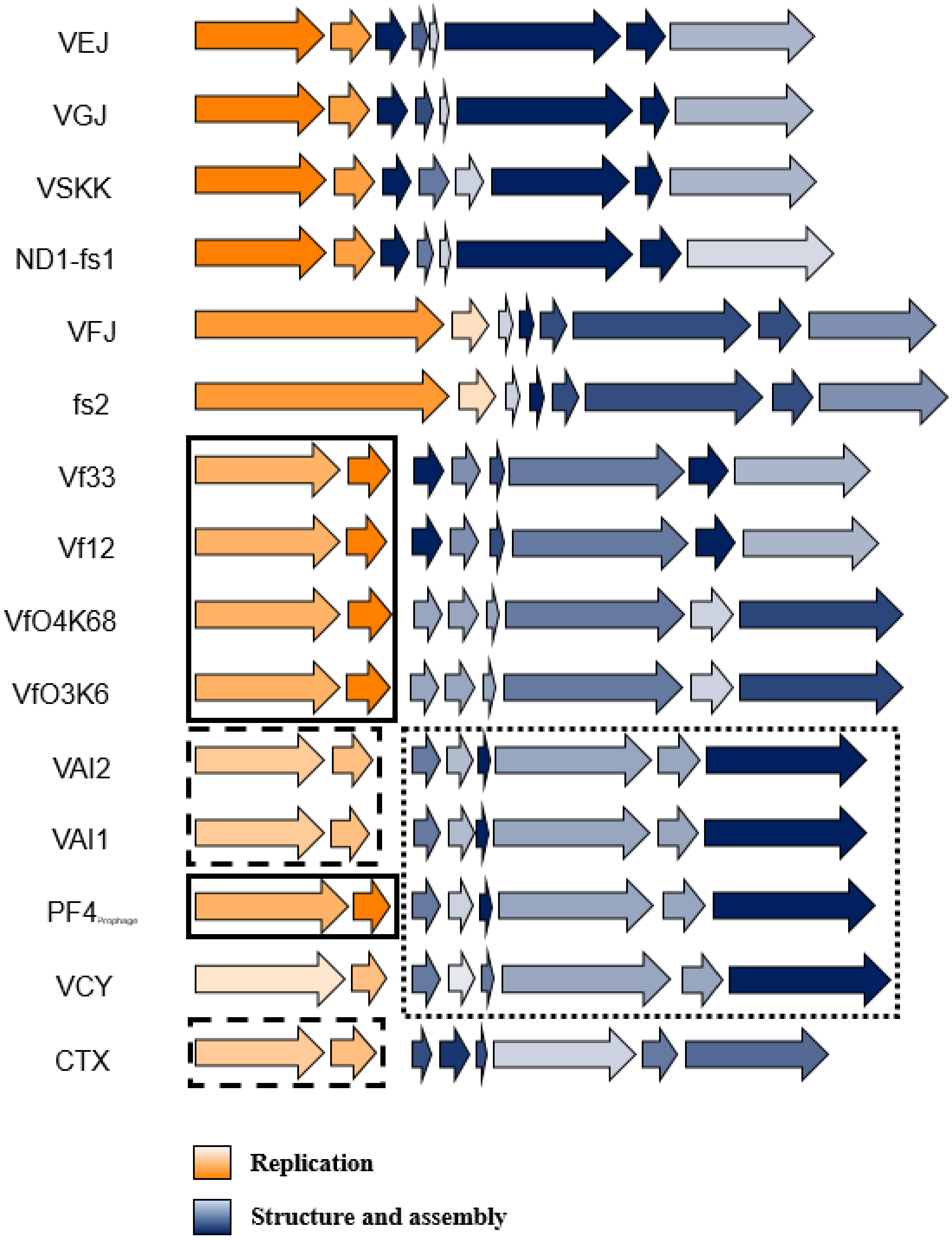

Figure S4. Mosaic genome structures among conserved genes in filamentous vibriophages. Open reading frames (ORFs) were colored according to their predicted function and the intensity of the color represents the respective ORF's relatedness to one another based on a phylogenetic tree (e.g. the first ORF of VEJ was closely related to similar ORFs of VGJ, VSKK and ND1-fs1 and most distantly related to VCY). The different frames mark observations related to this study (e.g. replication ORFs of the PF4 prophage were related to Vf33, Vf12, VfO4K68 and VfO3K6). The sequences of corresponding ORFs were aligned and used to construct separate ORF-specific phylogenetic trees using the maximum likelihood algorithm (100 bootstrapping). The prophage CON_phi2 was set as an outgroup to root each tree.

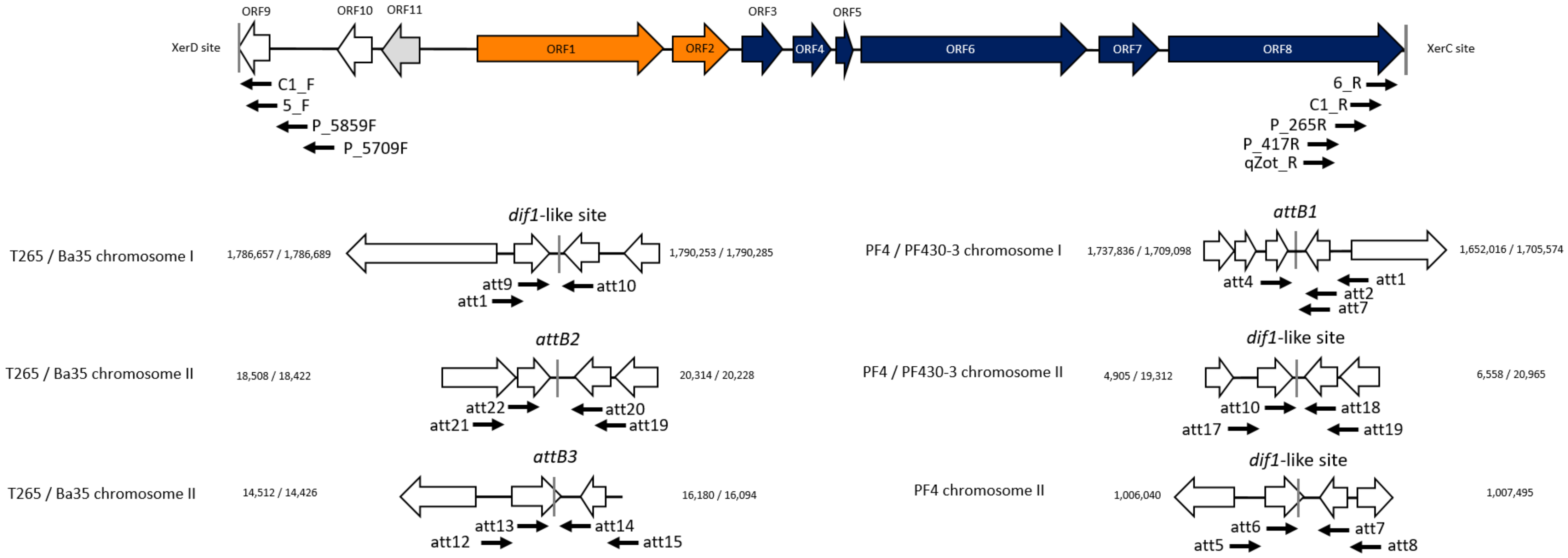

<u>Phage genome insertion PCR assays:</u>

Primer combination:	PCR result:
att1/att9/att10 + C1_F/5_F/P_5709F/6_R/C1_R/P_417R:	-
att19 + P_5709F/C1_F/5_F:	-
att19 + qZot_R/C1_R/6_R:	+
att22 + P_5709F/C1_F/5_F:	+
att22 + qZot_R/C1_R/6_R:	-
att12/att13 + P_5709F/P_5859F	+
att12/att13 + P_265R/P_417R:	-
att14/att15 + P_5709F/P_5859F	-
att14/att15+P_5709F/P_5859F	+

<u>Intact *attB* / *dif1*-like site PCR assays:</u>

Primer combination:	PCR result:
att1/att9 + att10:	+
att21/att22 + att20/att19:	+
att12/att13 + att14/att15:	+

<u>Intact *attB* / *dif1*-like site PCR assays:</u>

Primer combination:	PCR result:
att4 + att1/att2/att7:	+
att17/att10 + att18/att19:	+
att5/att6 + att8/att7:	-

<u>Phage genome insertion PCR assays:</u>

Primer combination:	PCR result:
att4 + C1_F/5_F:	+
att4 + 6_R/C1_R:	-
att1/att2/att7 + C1_F/5_F:	-
att1/att2/att7 + 6_R/C1_R:	+
att17/att10 + C1_R/P_417R	-
att17/att10 + P_5859F/P_5709F	-
att18/att19 + C1_R/P_417R	/*
att18/att19 + P_5859F/P_5709F	/*
att5/att6/att8/att7 + C1_F/5_F/6_R/C1_R	-

Figure S5. Schematic overview of the PCR analysis of VAIϕ genome insertions into *dif1*-like sites. Schematic overview of the primer locations on the targeted VAIϕ genome and bacterial chromosomes. Each primer to the left and right of the '+' were combined with one another to test for PCR amplification (e.g. att1 was combined with C1_F, 5_F, P_5709F, etc.). Successful and lack of product formation, as determined by gel electrophoresis, are denoted with a + or -, respectively. The denoted *attB* sites were interpreted as VAIϕ genome insertion sites and the *dif1*-like sites were not considered VAIϕ genome insertion sites due to inconsistent or lack of PCR product formation as shown. Open arrows represent ORFs oriented in the transcription direction and the color indicate the putative functions of structure-and-assembly, replication, regulation and other/hypothetical for blue, orange, gray and white, respectively. * / indicates inconsistent results of ≥6 independent repetitions.

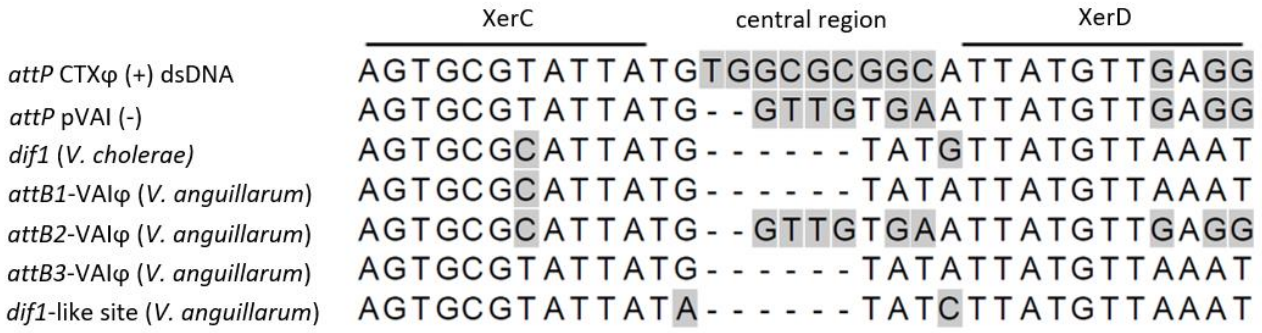

Figure S6. Alignment of phage and bacterial attachment sites with a *dif1*-like site. Alignment of CTXϕ and pVAIs *attP* plus *V. cholerae dif1* [39], *V. anguillarum attB* sites and a *dif1*-like site where VAIϕ genome insertion was not observed (Fig. 3). The *dif1*-like site contains two nucleotide changes at conserved positions in the central region. The extended lines indicate XerC and XerD binding sites and are separated by a central region. Vertical lines indicate alignment gaps and non-identical nucleotides are shaded.

Table S1. List of primer sequences and their purpose in this study.

Primer	Sequence	Purpose in this study
Zot_F	5’-GTTCCGTCTGGTGTTTTGGC-3’	Evaluate the presence of phage
Zot_R	3’-GCGCTCTCAATGTGTTAGCG-5’	Evaluate the presence of phage
qZot_F	5’-GTATGGCCCGTGTAGTACAAGT-3’	Estimation of *zot* quantity by qPCR and presence of (pro)phage
qZot_R	3’-TCAATCGTCCAGTGTCAGCA-5’	Estimation of *zot* quantity by qPCR and presence of (pro)phage
DnaJ_F	5’-GTGCGCTATCAAGTACAGGC-3’	Evaluate the presence of chromosomal DNA and qPCR
DnaJ_R	3’-TATGTCCCAAAGTAGGCCCG-5’	Evaluate the presence of chromosomal DNA and qPCR
1_R	3’-GCAAATAATGCGCACTCTGA-5’	Evaluate the presence of circularized prophage elements
2_R	3’-GCTTGCTCAAGCTGACAAAA-5’	Evaluate the presence of circularized prophage elements
3_F	5’-CAGCAATTGACCGGAGATTC-3’	Evaluate the presence of circularized prophage elements
4_F	5-’CAGGAACGAAAACCCAAGAA-3’	Evaluate the presence of circularized prophage elements
5_R	3’-GAACGCGCTGAAGTCAAACT-5’	Evaluate the presence of circularized phage elements and insertion of the VAIϕ genome in host chromosome
6_F	5’-AACGAAAGGACTCGCTTGTG-3’	Evaluate the presence of circularized phage elements
7_F	5’-TCCTAGCATGTACGCAGCAA-3’	Evaluate the presence of circularized phage elements and sequenced to determine the

		insertion of the pVAI1 genome in host chromosome
C1_F	5’-CAGAGGCAAACCCTCAACAT-3’	PCR products sequenced to close the genome of pVAI2 and insertion of the VAIϕ genome in host chromosome
C1_R	3’-GCGGCCAACGATATCAAATA-5’	PCR products sequenced to close the genome of pVAI2 and determine the insertion of the pVAI1 genome in host chromosome
C2_F	5’-GAACGCATCAAGCGGTATTT-3’	PCR products sequenced to close the genome of pVAI2
C2_R	3’-CCGCTTATGCTGCTTACTTTG-5’	PCR products sequenced to close the genome of pVAI2
C3_F	5-’GAGGCCGTCTTATAGGCAAA-3’	PCR products sequenced to close the genome of pVAI2
C3_R	3’-AGCCTGATTTGCTTGAGTCC-5’	PCR products sequenced to close the genome of pVAI2
C4_F	5’-CGAACCTAGCGAATTGCTTT-3’	PCR products sequenced to close the genome of pVAI2
C4_R	3’-TTGCCCGACACTTACAAACA-5’	PCR products sequenced to close the genome of pVAI2
C5_F	5’-GAAGACCAAGGACGTGGAGA-3’	PCR products sequenced to close the genome of pVAI2
C5_R	3’-GTCAGACCTTGCGTCTGTCA-5’	PCR products sequenced to close the genome of pVAI2
att1	5’-GGCATTTTCAACCGTCAACT-3’	Evaluate the presence of VAIϕ genome insertion
att2	5’-GGCCACGAATTCCTGAACTA-3’	Evaluate the presence of VAIϕ genome insertion
att3	3’-TCAGAGTGCGCATTATTTGC-5’	Evaluate the region adjacent to *dif1* of PF4-*zot*-prophage^{-} chromosome I
att4	3’-AAATTGTAGGCGTTGGGACA-5’	Evaluate the presence of VAIϕ genome insertion and sequenced to determine the insertion of the pVAI1 genome in host chromosome
att5	5’-GACCAGAGCGGCCTAATGTA-3’	Evaluate the presence of VAIϕ genome insertion
att6	5’-AGCACAACGGGCTAGGATTA-3’	Evaluate the presence of VAIϕ genome insertion
att7	TCCAACCAAACTCACCCAAT	Evaluate the presence of VAIϕ genome insertion and sequenced to determine the insertion of the pVAI1 genome in host chromosome
att8	3’-ACCACGGCGAAGTAGTGAAC-5’	Evaluate the presence of VAIϕ genome insertion
att9	5’-ACTCCAACGCCAAATTGTTC-3’	Evaluate the presence of VAIϕ genome insertion
att10	CAAGCTGATGCAATTGATGG	Evaluate the presence of VAIϕ genome insertion

att12	5’-AACGCATAAGCGATGTGGTT-3’	Evaluate the presence of VAIϕ genome insertion and sequenced to determine the insertion of the pVAI1 genome in host chromosome
att13	5’-GAGCTGGCTTTACTCGGATG-3’	Evaluate the presence of VAIϕ genome insertion
att14	3’-TTATGAGCGCAAATGAGTGC-5’	Evaluate the presence of VAIϕ genome insertion
att15	3’-GGCGAATTATCGACCGTTTA-5’	Evaluate the presence of VAIϕ genome insertion and sequenced to determine the insertion of the pVAI1 genome in host chromosome
att17	5’-GCTCAGCACTGGGAAAAATC-3’	Evaluate the presence of VAIϕ genome insertion
att18	3’-GCGGCAGAAATCAAGAAAAG-5’	Evaluate the presence of VAIϕ genome insertion
att19	3’-CGGATTTAGGCCGTATCAAA-5’	Evaluate the presence of VAIϕ genome insertion and sequenced to determine the insertion of the pVAI1 genome in host chromosome
att20	3-’GCGGCAGAACTCAAGAAAAG-5’	Evaluate the presence of VAIϕ genome insertion
att21	5-’TCTCTGTGCTTGAGCGAGAA-3’	Evaluate the presence of VAIϕ genome insertion
att22	5’-TTCCAACCGGTGCTAAGTTC-3’	Evaluate the presence of VAIϕ genome insertion and sequenced to determine the insertion of the pVAI1 genome in host chromosome
P_265R	3’- ACGGGCCATACAACAAAGAG-5’	Evaluate the presence of VAIϕ genome insertion
P_417R	3’-GCAATAAAGCGGATTCTTCG-5’	Evaluate the presence of VAIϕ genome insertion and Sanger seqeunced
P_5709F	5’-AAGCCCCATTGATGTGGTAA-3’	Evaluate the presence of VAIϕ genome insertion and sequenced to determine the insertion of the pVAI1 genome in host chromosome
P_5859F	5’- CACGCGCTCTCTTATCCCTA-3’	Evaluate the presence of VAIϕ genome insertion

Table S2. List of phages and prophage host sequences and their respective accession no. used in this study

Phage or prophage	Bacteria host	Source of host isolation	Genome size (bp)	Accession no.; genomic position of prophage	Reference
VEJϕ	*Vibrio cholerae*	Cholera patient, Madras, India 1993	6842	FJ904927	[65]
VGJϕ	*Vibrio cholerae*	Cholera patient, Calcutta, India 1993	7542	AY242528	[61]
VSK	*Vibrio cholerae*	Endemic areas, India and Bangladesh	6882	AF453500	[66]
VSKK	*Vibrio cholerae*	Endemic area, Bangladesh	6834	AF452449	[67]
ND1-fs1	*Vibrio cholerae*	Environmental water sample, Vietnam 2010	6856	AB572858	[68]
VFJϕ	*Vibrio cholerae*	Fujian, China 1997	8555	KC357596	[69]
fs1	*Vibrio cholerae*	Diarrhea patient, Bangladesh	6340	D89074	[70]
fs2	*Vibrio cholerae*	India	8651	AB002632	[71]
Vf33	*Vibrio parahaemolyticus*	Patient, Fukuoka, Japan	7965	AB012573	[48]
Vf12	*Vibrio parahaemolyticus*	Patient, Fukuoka, Japan	7965	AB012574	[48]
VfO3K6 / f237	*Vibrio parahaemolyticus*	Diarrhea patient, Japan	8784	AB043678	[28]
VfO4K68	*Vibrio parahaemolyticus*	Diarrhea patient, Japan	6891	AB043679	[72]
pVAI1	*Vibro anguillarum*	Diseased sockeye salmon, USA	6117	MN200778	This study
pVAI2	*Vibro anguillarum*	Diseased atlantic salmon, UK	6117	MN200777	This study
VCYϕ	*Vibrio cholerae*	Oyster Pond, Woods Hole, MA, USA	7103	JN848801	[30]
CTXϕ	*Vibrio cholerae*	El Tor biotype isolate, Bangladesh and India	6969	KF664579 and HQ224500*	[13]
KSF-1ϕ	*Vibrio cholerae*	Surface water, Dhaka, Bangladesh	7107	AY714348	[50]

PF4 *zot*-encoding prophage	*Vibrio anguillarum*	Diseased atlantic salmon, Chile 2005	8960	CP010080/CP010081; 3,130,438 to 3,139,397 on chromosome I and 1,012,669 to 1,021,628 on chromosome II	[26]
Prophage CON_phi2	*Campylobacter concisus*	Human faeces	~12000	NC_009802.2; 1,581,886 to ~1,593,000	[73]

*Accession no. KF664579 was primarily used, however, the ORFs encoding *ctxA* and *ctxB* were selected from HQ224500 and used for Figure 2A.

Table S3. Annotation of pVAIs' ORFs

ORF	Strand	Size (aa)	Homologous protein	Homolog identity (%)	E-value	Signal or TM*	Predicted function**
ORF1	+	361	RstA (Vibriophage CTX)	75	0	-	Replication initiation
ORF2	+	112	RstB (Vibriophage CTX)	44	6,00E-30	-	Required for integration and replication
ORF3	+	76	Hypothetical (Vibriophage VCY)	73	9,00E-39	TM (?) ***	Structural and assembly protein
ORF4	+	72	Hypothetical (Vibriophage VCY)	45	3,00E-13	S, TM	Major coat protein
ORF5	+	32	ORFIV (Vibriophage KSF-1)	53	3,00E-07	TM	Unknown
ORF6	+	437	Minor capsid (Vibriophage VCY)	27	3,00E-29	S, TM	Receptor-binding protein
ORF7	+	114	ORFVIII (Vibriophage KSF-1)	56	5,00E-43	TM	Structural protein
ORF8	+	454	Zot-like (Vibriophage VCY)	64	0	TM	Assembly protein
ORF9	-	61	Hypothetical (*Vibrio anguillarum*)	93	2,00E-23	-	Unknown
ORF10	-	68	Hypothetical (*Vibrio cholerae*)	74	4,00E-09	-	Unknown
ORF11	-	64	RstR (Vibriophage CTX)	95	3,00E-40	-	Transcriptional regulator

ORFs were predicted using ORF finder and homology search was performed using the Blastp algorithm against the the *Inovirus* (taxid:10861) and *Inoviridae* (taxid:10860) databases (June,

2020). If no significant hits were retrieved, the ORFs were searched against the non-redundant database.
*Presence of signal peptide indicated with ‘S’ and a transmembrane domain designated with ‘TM’ as predicted by SignalP, TMHMM and Phobius.
**Predicted function was based on gene synteny and size, homology search, membrane topology, presence of signal peptide and subcellular localization.
*** Transmembrane domain was only predicted by Phobius in the C-terminus and not by TMHMM.

Table S4. ORF8 of pVAIs searched against the *Inovirus* (taxid: 10861) and *Inoviridae* (taxid: 10860) databases.

Homologous protein	Coverage (%)	E-value	Homolog identity (%)	Accession no. of homolog
Zot-like protein (*Vibrio* phage VCY)	100	0	64	YP_004934230.1
Zot-like protein (*Vibrio* phage KSF1)	100	0	67	YP_087078.1
Hypothetical protein (*Vibrio* phage VALG-phi6)	95	2,00E-106	38	QGT53739.1
Vpf461 (*Vibrio* phage VfO3K6)	95	3,00E-106	39	NP_059537.1
Zot (*Pseudomonas* phage pf8)	66	2,00E-22	27	QGZ15337.1
hypothetical protein Pf1p07 (*Pseudomonas* virus Pf1)	63	1,00E-21	28	NP_039606.1
Zot (*Vibrio* virus CTX)	71	2,00E-21	26	AWD92950.1
Zot (*Vibrio* virus CTX)	71	3,00E-21	25	AAL09680.1
Zot (*Vibrio* phage pre-CTX)	73	4,00E-21	25	AKF12299.1
Zot (*Vibrio* phage pre-CTX)	73	4,00E-21	26	ALF99877.1
Zot (*Vibrio* virus CTX)	73	4,00E-21	26	ACV73683.1
Zot (*Vibrio* virus CTX)	71	4,00E-21	25	AAL09684.1
Zot (*Vibrio* virus CTX)	73	5,00E-21	26	YP_004286239.1
Zot (*Vibrio* virus CTX)	71	6,00E-21	25	ADE80688.1
Zot (*Vibrio* phage pre-CTX)	72	6,00E-21	27	ALM30804.1

Zot (*Vibrio* virus CTX)	71	6,00E-21	25	AAL09690.1
Zot (*Vibrio* virus CTX)	71	8,00E-21	25	AHY24960.1
PA0726 (*Pseudomonas* virus Pf1)	65	1,00E-20	27	AAQ94688.1

Table S5. **Annotation of ORF9 before and after integration in *Vibrio anguillarum* genomes**

ORF9 before and after integration	Size (aa)	Homologous protein	Homolog identity (%)	E-value	TM*
ORF9^{pVAI}	61	Hypothetical (*Vibrio quintilis*)	68	1,00E-13	-
ORF9^{integrated} in *attB1*	118	Hypothetical (*Vibrio quintilis*)	76	1,00E-12	TM
ORF9^{integrated} in *attB2*	95	Hypothetical (*Vibrio quintilis*)	72	4,00E-13	-
ORF9^{integrated} in *attB3*	184	Hypothetical (*Vibrio quintilis*)	72	2,00E-11	TM (?) **

ORFs were predicted using ORF finder and homology search was performed using the Blastp algorithm against the non-redundant database (June, 2020).
*Presence of a transmembrane domain designated with 'TM' as predicted by TMHMM and Phobius.
**Transmembrane domain was only predicted by Phobius in the C-terminus and not by TMHMM.
